# A patient recall program to enhance decisions about prostate cancer screening: A feasibility study

**DOI:** 10.1186/1471-2296-10-75

**Published:** 2009-11-30

**Authors:** Thomas D Denberg, Manisha Bhide, Alyssa Soenksen, Trina Mizrahi, Laura Shields, Chen-Tan Lin

**Affiliations:** 1 Department of Medicine, University of Colorado Denver School of Medicine, Aurora, Colorado, USA; 2 Colorado Health Outcomes Program, University of Colorado Denver School of Medicine, Aurora, Colorado, USA

## Abstract

**Background:**

Lack of time and competing demands limit the ability of patients and providers to engage in informed decision-making discussions about prostate cancer screening during primary care visits. We evaluated a patient recall invervention to mitigate these challenges.

**Methods:**

Using mail and telephone outreach we invited men age 50-74 years without a PSA test in the prior 12 months to make appointments with their primary care providers in order to discuss the pros and cons of PSA-based prostate cancer screening. We assessed patient responsiveness to the program, provider documentation of screening discussions, orders for PSA laboratories, and provider attitudes.

**Results:**

Out of 80 eligible patients, 37 (46%) scheduled and 28 (35%) completed a recall appointment. A large majority (91%) of patients eligible for PSA screening received an order for this test. Providers documented PSA discussions more often for these patients than for a recent sample of their other patients who received traditional care (47.8% vs. 12.5%, p = 0.009). Twelve of 14 participating providers felt the program improved their ability to impart information about the risks and benefits of screening, but were uncertain whether it influenced their patients' preexisting preferences for screening. Some expressed doubts about the advisability of PSA-specific appointments.

**Conclusion:**

To a limited extent, this pilot recall intervention enhanced opportunities for discussions of prostate cancer screening between patients and their primary care providers. As currently configured, however, this program was not found to be feasible for this purpose. A future version should promote screening discussions in the context of a broader range of health maintenance concerns and include more detailed, low-literacy information to educate patients in advance of clinic visits.

## Background

Prostate cancer is the second-leading cause of cancer death in men over the age of 50. The prostate specific antigen (PSA) test combined with a digital rectal exam (DRE) can detect prostate cancer early; however, there is little high-quality evidence that screening and current treatments, many of which have disabling side effects, reduce mortality [1]. For this reason, professional organizations including the American Cancer Society and the United States Preventive Services Task Force (USPSTF) recommend that before proceeding with screening, men have an informed discussion with their health care providers regarding the risks and benefits of the PSA test [2]. Unfortunately, this recommendation is extremely challenging to implement [3]. The issues and terminology are complex and frequently difficult for patients to understand [4]. Often, providers have time to focus only on acute concerns during clinic visits, referring patients for a PSA test without a discussion of its pros and cons [5]. In addition, providers who are unaware of screening guidelines may assign limited importance to informed decision-making or may be inexperienced with such discussions [6].

In response to these challenges, efforts have focused on educating and activating patients for discussions through the use of decision aids and other educational materials. A few studies have demonstrated that decision aids are associated with increased knowledge and reduced screening rates [7,8]. Personnel and space constraints, however, limit the ability of many medical practices to deliver these materials to patients immediately prior to office visits. In addition, because of time pressures and uncomfortable accommodations, the clinic setting may not be an optimal environment for learning.

Here, we evaluate a modified approach to informed decision-making for prostate cancer screening. The approach involves using a registry to identify patients eligible for PSA screening and proactively outreaching to these patients by mail and telephone in order to schedule appointments specifically intended to facilitate meaningful discussions with medical providers about the risks and benefits of screening. The goal is to give patients the opportunity to consider a brief educational message at leisure in their home environment while providing ample time in clinic to discuss PSA screening with medical providers. We assessed the operational feasibility of the program; level of patient willingness to schedule and complete decision-making appointments; documentation of PSA screening discussions during clinic visits; rates of PSA ordering; and provider feedback about the program.

## Methods

### Study Setting

This study was conducted in a large ambulatory primary care practice affiliated with the University of Colorado Hospital (UCH). The practice provides primary care to a diverse patient population (approximately 50,000 visits per year) and is staffed by 40 attending physicians, four nurse practitioners, and 20 primary care residents. Attending providers were consulted and agreed ahead of time to participate in the pilot program described below.

### Patient Population

Based on automated electronic health record (EHR, Allscripts Touchworks, version 10, Chicago, IL) abstraction, men were deemed to meet initial inclusion criteria if they were age 50 to 74 years, had an attending (rather than resident) primary care provider (PCP), had seen a PCP in the practice at least once in the preceding 18 months (to minimize the inclusion of patients no longer receiving care in the system), and did *not *have an administrative claim for a PSA test within the past 12 months (to coincide with standard recommendations for screening on a yearly basis *if *screening is to be carried out). Through a subsequent manual review process, men from this initial sample were excluded from outreach if they had clinic notes indicating an active cancer or a terminal diagnosis; were deceased; or no longer appeared to be receiving care within the system. In addition, men were excluded if PSA testing would not be warranted for *screening *purposes because of a history of prostate cancer or a previously elevated PSA result or prostate nodule. In other words, it is often not possible to determine eligibility based on automated (computer-based) EHR-review alone; a manual review of the EHR as well as patient return postcards and telephone contact are often necessary to identify patients who have received PSA screening outside the system in the specified time frame (12 months), who are no longer receiving care in the system (more likely if they inform us of same or have not received care in the system in >18 months), and who are ineligible for PSA *screening*. Such patients, once identified, are deemed ineligible and not offered phone-based appointment scheduling.

### Intervention Description and Implementation

Registry records of men meeting initial inclusion criteria were imported into an information management utility developed for this and other preventive and chronic disease outreach interventions [9-12]. The presence of exclusionary criteria was determined for a randomly-selected sample of these men by means of manual review of the electronic health record. This process proceeded until a final sample of between 100 and 120 men was identified as eligible for outreach. Based on prior experience, we estimated that 25-30% of these men would subsequently be deemed ineligible for participation in the program based on information obtained through the outreach process and, accordingly, would not be offered appointment scheduling over the phone (e.g. no longer receiving care in the system, received PSA screening outside the system, deceased). The final eligible sample, estimated to include 75-100 men, would be sufficient for assessing the operational feasibility of the program.

The information management utility generated invitation letters to be mailed to each patient's home. Each letter, bearing the name of the patient's PCP on the signature line, included the following message: "The PSA test can help doctors find and treat prostate cancer at an early stage, but the test is not perfect and does have potential downsides. Believe it or not, for example, there is still a lack of evidence that prostate cancer screening saves lives. Also, this type of screening can sometimes lead to unnecessary treatments that cause permanent side effects. Cancer prevention experts recommend that men speak with their doctors about the PSA test before going ahead with screening. The goal of a visit with your primary care provider is not to get prostate cancer screening but to help you make an informed decision about *whether *to get prostate cancer screening." The letter encouraged the patient to contact our call center to schedule a PCP appointment to discuss PSA screening. A postage-paid return postcard accompanied each letter on which the patient could indicate whether he had recently had a PSA test outside of the health system, no longer received primary care within the system, was uninterested in discussing PSA screening, or preferred to be contacted at a specified time and telephone number. If the patient did not respond to the letter within two weeks by postcard or phone, an outreach coordinator (OC) made up to three calls to his home at various times of day, leaving a voice message on the first and last attempts. If a patient could not be reached after three calls had been made, he was considered a passive decliner of a PSA screening discussion.

If telephone contact was established, the OC reviewed the rationale for having a discussion about PSA screening (i.e. men should know the pros and cons of prostate cancer screening before making a decision and the PCP will be able explain these). If the patient accepted, the OC scheduled a 20-minute appointment over the telephone. The OC then sent a brief note to the PCP via the EHR summarizing the purpose of the upcoming visit: "Patient X has scheduled an appointment with you at date/time to discuss prostate cancer screening. As you know, there are benefits and risks to such screening and it is important for men to be informed before making this decision. An informed decision-making reference is available at the following website: <url>" [13]. Finally, the OC mailed a reminder postcard to the patient noting the date, time, and location of the appointment. The call center was staffed between the hours of 8:00 am and 6:00 pm, Monday through Friday; at other times patients could leave messages requesting a callback. The outreach intervention was conducted between August 1 and September 9, 2008.

### Primary Outcomes

We assessed the following outcomes:

#### Appointment scheduling and completion

These were characterized as the proportions of eligible participants who, during and after mail and telephone outreach, scheduled and completed an appointment with a PCP to discuss prostate cancer screening. Only those patients who scheduled an appointment with an OC through the call center (as opposed to the usual clinic scheduling line), and who received verbal information from an OC about the informed decision-making purpose of the clinic visit, were considered to be responsive to the program.

#### Provider documentation of prostate cancer screening discussions and comparisons with traditional care

We determined the proportion of patients whose providers documented PSA screening discussions in the EHR during appointments scheduled for this purpose. Two research assistants also independently characterized the content of the clinic notes according to the presence or absence of three types of elements: (1) *PSA discussion *- generic reference to or specific details evidencing at least some screening "discussion" or "conversation"; (2) *At least one downside *- generic reference to having discussed, or specific documentation of, at least one downside of PSA screening; and (3) *Patient preference *- documentation that the patient did or did not wish to proceed with screening. For each provider who had at least one patient that completed a recall visit for the purpose of a screening discussion, we carried out a similar review of EHR documentation for the three most recent patients for whom the same provider completed PSA screening during a period immediately preceding implementation of the intervention. Based on USPSTF guidelines, these patients should (ideally) have engaged in a decision-making discussion before completing screening, which should have been documented in the EHR. We regarded these patients as having received traditional care. In other words, theoretically for this group, decision-making discussions could have taken place opportunistically during traditionally-arranged, episodic clinic visits (as opposed to clinic visits scheduled specifically for the purpose of carrying out such discussions). We then compared traditional and intervention care in terms of the presence of each type of documentation element.

#### PSA ordering

The rate of PSA ordering was characterized as the proportion of screen-eligible patients whose providers ordered or indicated an intention to order a PSA screening test during the clinic visit.

#### Provider Feedback

We devised a brief online survey which we administered after the intervention to participating providers in order to assess their open-ended opinions about their willingness to discuss PSA screening with patients; the value they place on such discussions; the mechanics of the intervention; and perceived effects on quality of care.

### Statistical Methods

Statistical procedures were performed using STATA (version 8.0, College Station, TX). Chi-square tests were used to determine the strength of association between patient characteristics (age, race/ethnicity, marital status, and history of prior PSA screening in the health system) and willingness to schedule an appointment. Non-parametric Mann-Whitney tests were used to derive aggregate comparison measures of participating providers in terms of their average rates of including EHR documentation of screening discussion elements during traditional care and intervention-related visits. Descriptive statistics and quotes illustrative of recurrent themes were used to characterize the results of the provider survey.

### Institutional Review Board

This intervention was designed and carried out as a quality improvement research program that relied on standard methods for creating patient registries and providing patient outreach. The Colorado Multiple Institution Review Board approved publication of results following the removal of personal health information.

## Results

Of the 154 patients who met initial, automated inclusion criteria, 40 (26%) were identified as meeting one or more exclusion criteria after brief manual review, leaving 114 in outreach sample. Characteristics of 80 patients deemed eligible for program participation after outreach was completed are summarized in Table 1. Of these, 37 patients (46%) scheduled an appointment through the call center. In this relatively small sample, patient characteristics were not associated with a willingness to schedule an appointment. Of 15 patients who, in responding to outreach, said they were not interested in making an appointment, 8 reported that they had previously had a decision-making discussion with their PCP and did not want to complete screening. However, no patient said he declined to schedule an appointment *because *of information contained in the outreach materials.

**Table 1:** Patients eligible for PSA screening discussion and proportion scheduling a provider visit for this purpose (n = 80)

Characteristic	N (%)	N (%) that scheduled appointment*
Age		

50-64	60 (75%)	32/60 (53%)

65+	20 (25%)	5/20 (25%)

		

Marital Status		

Married	58 (73%)	28/58 (48%)

Not Married	19 (24%)	8/19 (42%)

Unknown	3 (4%)	1/3 (33%)

		

Race/ethnicity		

White	42 (53%)	20/42 (48%)

Black	12 (15%)	7/12 (58%)

Other/Unknown	26 (32%)	10/26 (38%)

		

History of PSA test at UCH^†^		

Yes	41 (51%)	22/41 (55%)

No	39 (49%)	15/39 (39%)

		

TOTAL	80	37/80 (46%)

* Chi-square *p *was non-significant across all measured characteristics.

^† ^UCH = University of Colorado Hospital and Clinics.

**Figure 1:**
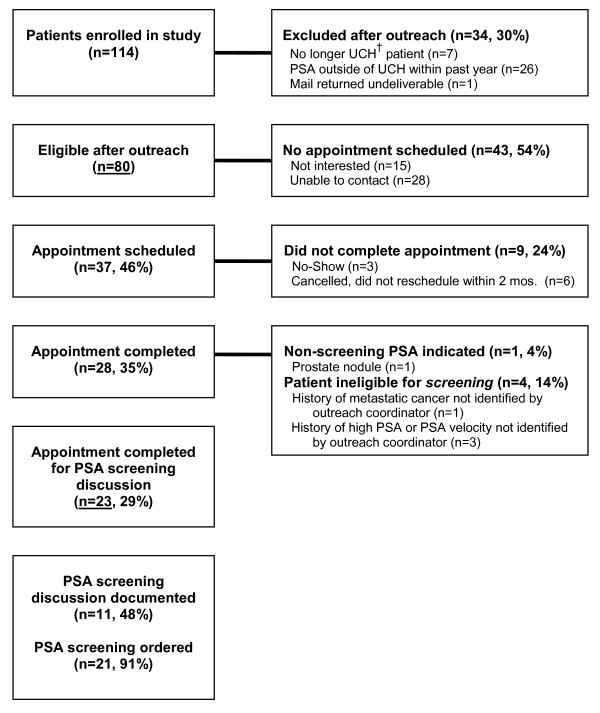
**Patient and provider responsiveness to recall intervention**. ^†^UCH = University of Colorado Hospital and Clinics.

Patient and provider responsiveness at various points during the intervention process are summarized in Figure 1. Ultimately, 23 patients truly eligible for prostate cancer screening completed an appointment with one of 14 PCPs. Twenty-one (91%) of these patients received an order for a PSA test. Only one patient declined PSA screening as a result of a discussion with his provider. In another instance, the patient and provider appear to have focused on issues other than prostate cancer screening. In comparison with traditional care provided by the same PCPs, patients with intervention-related visits were several times more likely to have clinic notes that explicitly documented a discussion about screening and that made reference to at least one downside of screening. Meanwhile, there was no significant difference in the presence of documentation regarding the *patient's *final screening preference (Table 2).

**Table 2:** Presence of informed decision-making documentation: traditional care vs. intervention^†^

Documented element*	Traditional care	Recall intervention	*p*
PSA discussion	12.5%	47.8%	*0.05*

At least one downside	5.0%	43.5%	*0.01*

Patient preference	20.0%	43.5%	*0.09*

^† ^Mann Whitney-U test, number of medical providers = 14.

* *PSA discussion*: Generic reference to or specific details evidencing at least some "discussion." *At least one downside*: Generic reference to having discussed, or specific documentation of, at least one downside of PSA screening. *Patient preference*: Documentation that the patient does or does not wish to proceed with screening.

Thirty-seven attending PCPs (93% of the total sample) who responded to the survey, including 14 that participated in intervention-related visits, agreed that discussions of the risks and benefits of screening are somewhat to very important. At the same time, however, two-thirds of respondents reported that such discussions are very challenging and rarely take place, even when extra time is made available. Said one provider, "Competing demands, time, and complexity of working through the hypotheticals are probably the biggest challenges. The near universal message in the media that men should have prostate screening also presents a challenge." Twelve of 14 PCPs who participated in intervention-related visits felt that their ability to share information about screening was enhanced, but most remained uncertain whether the extra time and information did much to sway their patients' *a priori *preferences. Primarily because of problems with patient access, a third of all respondents felt that prostate cancer screening and associated discussions should not be separated out from a longer annual exam in which other preventive health concerns are addressed. Finally, three-fourths of respondents were willing to let a mid-level provider (physician's assistant or nurse practitioner) carry out prostate cancer screening discussions on their behalf, but one detractor suggested that this would only "increase the level of fragmentation" in patient care.

## Discussion

Given the uncertain benefits and risks of harm associated with PSA-based screening, patients should participate in an informed decision-making process about this test [14]. However, lack of comprehension of common prostate cancer terms, especially among low-literacy populations, as well as a lack of time during clinic visits, are key barriers to high-quality discussions [4,5]. Accordingly, we evaluated a patient recall intervention designed to activate patients and PCPs for discussions about the pros and cons of prostate cancer screening and to provide sufficient time for carrying out such discussions. Approximately 30% of eligible patients completed clinic visits for this purpose, a respectable rate that is commensurate with what we have obtained using a very similar intervention for other preventive services [11,12]. Second, providers believed that, despite having an uncertain influence on patient preferences, the program helped to improve the process of educating patients. Finally, for these patients, screening discussions were documented much more frequently than for patients who completed screening through traditional care. In these ways, the program was found to be operationally feasible.

In a larger sense, however, it remains unclear whether the pilot program achieved its key objective, which is to help patients make genuinely informed decisions about screening. First, whereas we would have hoped that documentation of a discussion would have been evident in all of the intervention clinic visit notes, such documentation was present in less than half of these. Second, it is doubtful that all patients responded with equal enthusiasm to the offer of a *conversation *about screening. For example, patients whose primary motivation was to complete screening might have been more likely to schedule appointments *in order *to complete screening. For these patients, discussions may have done little to change their minds (we observed, for example, that PCPs ordered a PSA test for almost all patients who completed visits). Meanwhile, patients with little interest in screening at the time of outreach, or after receiving a letter, might not have found the offer of a discussion particularly compelling and therefore may have declined to schedule one. On the other hand, we noted that half of these patients declined to schedule a visit because they had already made a decision to forego screening based on a prior conversation with their PCP about the topic. This supports the idea that such discussions can have an important influence on patient attitudes and behavior. A key question, then, is whether a patient recall program can motivate appointment scheduling among patients with ill-defined preferences for PSA screening.

These considerations, in addition to provider feedback about access problems in the clinic, suggest that a future recall program would be more effective if it facilitated PSA discussions within the context of addressing a larger set of health maintenance concerns. We did not wish to overwhelm patients with written information about prostate cancer screening, but earlier work suggests it may nonetheless be beneficial to include with the outreach materials more robust, low-health literacy educational materials or a PSA decision aid as these could help patients focus and be more prepared for discussions when they finally take place [15].

We remain intrigued by a patient-centered medical home model of team-based care in which - instead of physicians - mid-level providers, including nurse practitioners and physician's assistants, assume greater responsibility for the provision of primary prevention services [16]. With proper training and experience, these providers could become adept at leading patients through the challenges of an informed decision-making process. Educating patients about this arrangement and ensuring effective care coordination, however, would be necessary in order to overcome legitimate concerns about fragmented care.

While this study offers lessons for future work in prostate cancer screening informed-decision-making, it does have important limitations. The intervention included a relatively small number of patients, precluding meaningful subgroup analysis, and was performed in a single academic healthcare setting that is not representative of other types of practice environments. We did not assess patient knowledge, screening preferences, decision-making locus, and satisfaction before or after clinic visits. The lack of documentation of PSA discussions among some patients in the intervention group does not mean that such discussions did not take place. Similarly, it was not practically feasible to identify patients receiving traditional care who discussed PSA screening and whose providers, as a consequence, did *not *order a PSA test. Nonetheless, we believe that where PSA tests *were *ordered, the intervention and traditional care differences in documentation are significant and likely to reflect meaningful differences in the frequency and quality of the decision-making process. Future versions of this intervention should improve accuracy in the eligibility review process (e.g. better identification and exclusion of patients with previously-elevated PSA results) as well as accord greater attention to variables that are likely to influence cost-effectiveness and long-term sustainability (e.g. costs of staffing, increases in clinic visit volume).

Despite these limitations, this work had some strengths. First, under a low-risk clinical quality improvement umbrella, we were able to avoid selection bias by extending outreach to all men who were eligible for PSA screening rather than limiting participation to patients who formally consented to participation in a study. In contrast, prior studies of interventions to improve informed decision-making have limited participation to patients who provide written informed consent ahead of time [17,18], who have internet access [17], who are already scheduled for a PCP visit [8,15,17,18], or who are seeking a free PSA test [19]. Second, with rare exceptions [15,20], few prior studies have determined whether provider-patient discussions actually took place and have instead measured only patient *intentions *to be screened rather than provider orders for PSA tests. Finally, a recent systematic review indicates that only a very small number of interventions to educate and activate patients have been administered outside of clinical settings [7].

## Conclusion

In summary, this patient recall intervention appeared to activate and enhance opportunities for discussions of prostate cancer screening among male patients and their primary care providers, although not as frequently as desired. As currently configured, we cannot recommend this recall program for prostate cancer or other controversial screening tests. This type of intervention may be of greater interest and acceptability to patients if a future version promotes screening discussions in the context of a broader range of health maintenance activities and includes more detailed, low-literacy information designed to educate patients in advance of clinic visits.

## Abbreviations

DRE: digital rectal examination; EHR: electronic health record; OC: outreach coordinator; PCP: primary care provider; PSA: prostate specific antigen; USPSTF: United States Preventive Services Task Force.

## Competing interests

The authors declare that they have no competing interests.

## Authors' contributions

TDD conceived the study, supervised intervention implementation and evaluation, and assumed primary responsibility for manuscript preparation. CTL and MB assisted in planning the intervention, reviewing the results, and preparing the final manuscript. AS, TM, and LS carried out the intervention and data collection. TM also completed the statistical analysis. All authors read and approved the final manuscript.

## Note

Supported in part by funding by The American College of Physicians Foundation Health Literacy Awards Program. (Denberg, PI) and the American Cancer Society, MRSG-06-081-CPPB (Denberg, PI).

## Pre-publication history

The pre-publication history for this paper can be accessed here:

http://www.biomedcentral.com/1471-2296/10/75/prepub
